# Fucoidan from *Pearsonothuria graeffei* alleviates dyslipidemia in ApoE^−/−^ mice by regulating reverse cholesterol transport and promoting bile acid synthesis

**DOI:** 10.3389/fnut.2026.1974149

**Published:** 2026-10-19

**Authors:** Ting Zhao, Yang Yu, Yin Wang, Liyang Zhang, Junqi Xiao, Shiguo Chen, Shan Li

**Affiliations:** 1 Department of Clinical Nutrition, Affiliated Hospital of Qingdao University, Qingdao University, Qingdao, China; 2 Department of Cardiology, The Central Hospital of Wuhan, Tongji Medical College, Huazhong University of Science and Technology, Wuhan, China; 3 Institute of Nutrition & Health, School of Public Health, Qingdao University, Qingdao, China; 4 College of Biosystem Engineering and Food Science, Zhejiang University, Hangzhou, China

**Keywords:** atherosclerosis, bile acid, fucoidan, lipid metabolism, reverse cholesterol transport

## Abstract

**Background:**

The fucoidan extracted from sea cucumber *Pearsonothuria graeffei* (Fuc-*Pg*) has beneficial anti-inflammatory and lipid-lowering effects, suggesting its potential anti-atherosclerotic properties.

**Methods:**

ApoE^−/−^ mice were fed a high-fat diet (HFD) for 12 weeks. Fuc-*Pg* (20 mg/kg and 80 mg/kg) was administered by oral gavage once daily.

**Results:**

The results indicated that Fuc-*Pg* intervention reduced aortic plaques, suppressed systemic inflammation, and alleviated dyslipidemia in ApoE^−/−^ mice. Mechanistically, Fuc-*Pg* reduced lipid absorption by decreasing CD36 and microsomal triglyceride transfer protein (MTP) expression levels in the small intestine, enhanced reverse cholesterol transport (RCT) by increasing hepatic LDLR and ABCG5, and facilitated bile acid synthesis by increasing CYP7A1 and CYP27A1 expression. Increased fecal lipid and hepatic bile acid levels further confirmed that Fuc-*Pg* exerted preventive effects in ApoE^−/−^ mice by reducing lipid absorption.

**Conclusion:**

Fuc-*Pg* alleviated atherosclerosis by modulating cholesterol metabolism and reducing lipid absorption. This study elucidates the nutritional application of fucoidan as a functional food ingredient and explores its potential mechanism.

## Graphical abstract

**Figure:**
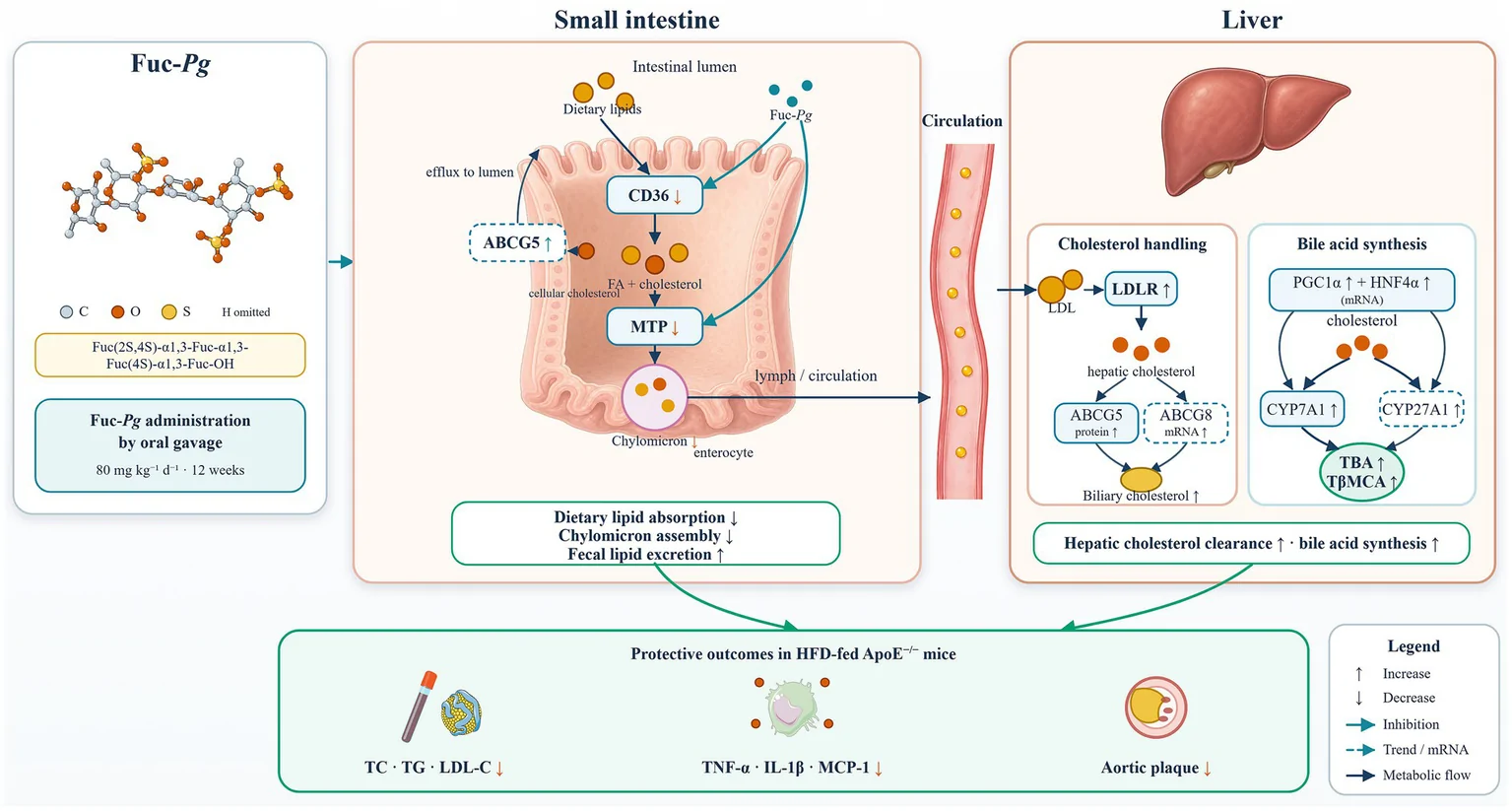
Possible mechanisms by which Fuc-Pg improves lipid metabolism in ApoE^−/−^ mice fed an HFD. Fuc-Pg, fucoidan from *Pearsonothuria graeffei*; HFD, high-fat diet.

## 1 Introduction

Atherosclerosis (AS) is a leading cause of cardiovascular and cerebrovascular diseases, such as ischemic stroke, which can seriously impact an individual’s physical and mental well-being (1). Hypercholesterolemia and chronic inflammation are recognized as major pathogenic factors in the development of AS (2). Prolonged consumption of a high-fat diet (HFD) leads to a sustained increase in circulating triglyceride-rich lipoproteins (TLRs). These TLRs accumulate within the vascular endothelium, eliciting early inflammatory responses that stimulate endothelial cells to secrete chemokines. These chemokines facilitate the recruitment of adherent leukocytes to the arterial intima, where monocytes differentiate into macrophages. These macrophages subsequently phagocytose lipids and transform into foam cells, a critical indicator of atherosclerotic lesions (3). Currently, the predominant treatment strategy for atherosclerosis is lipid-lowering therapy, which primarily involves the use of statins, ezetimibe, and proprotein convertase subtilisin/kexin type 9 (PCSK9) inhibitors. However, these drugs exhibit certain side effects and limitations. Drugs such as statins may impose additional burdens on the liver. The lipid-lowering effect of ezetimibe is limited (4). Therefore, developing reliable strategies for preventing atherosclerosis is of great significance.

Fucoidan is mainly composed of fucose with sulfated groups and is derived from marine algae and invertebrates. It has been widely demonstrated to exhibit non-toxicity, minimal irritation, and a variety of health-beneficial biological activities (5). It is a promising candidate for applications in functional foods and the pharmaceutical industry due to its anti-inflammatory, lipid-lowering, and immune-modulating activities (6). Fucoidans have been reported to decrease serum lipids both in animal models and human studies (7, 8). Fucoidan from brown seaweeds has anti-atherosclerotic activity by preventing oxidized low-density lipoprotein (oxLDL)-mediated foam cell formation in macrophages (9). As a kind of polysaccharide, the absorption of fucoidan is limited due to its high molecular weight (10). In addition, studies indicate that orally administered fucoidan can be absorbed through the intestinal tract into systemic circulation, albeit with a relatively low absorption rate (10, 11). In one study, fluorescein isothiocyanate (FITC)-labeled fucoidan was administered via a tail vein injection, revealing a significant increase in fucoidan concentration in the liver, which suggests that the liver might also be a target organ for fucoidan (12). Our previous studies have shown that the intestine might be the primary target organ where fucoidan from sea cucumber *Pearsonothuria graeffei* (Fuc-*Pg*) exerts its hypolipidemic activity. Fuc-*Pg* might improve the lipid profile by regulating intestinal lipid metabolism, mitigating liver and colonic inflammation, and reshaping the gut microbiota (13, 14). The anti-atherosclerotic activity of Fuc-*Pg* has not yet been fully elucidated.

Therefore, we hypothesized that fucoidan might be instrumental in preventing AS by regulating lipid metabolism in both the intestine and liver. An AS model was established in ApoE^−/−^ mice fed an HFD. The effects of Fuc-*Pg* on lipid transport in the liver and gut were investigated to explore the potential pathways. Additionally, cholesterol conversion in the liver was also investigated. These findings provide new insights into the mechanisms by which fucoidan exerts anti-atherosclerotic activity.

## 2 Materials and methods

### 2.1 Materials

Simvastatin was purchased from Aladdin (Shanghai, China). Detection kits for triglyceride (TG), cholesterol (TC), low-density lipoprotein cholesterol (LDL-C), high-density lipoprotein cholesterol (HDL-C), and total bile acid (TBA) were procured from Nanjing Jiancheng Bioengineering Institute (Nanjing, China). Enzyme-linked immunosorbent assay (ELISA) kits for tumor necrosis factor-α (TNF-α), interleukin-1β (IL-1β), and monocyte chemoattractant protein-1 (MCP-1) were purchased from Jingmei Biotechnology Company (Jiangsu, China). Information on bile acid standards was provided in Supplementary Table 1. Sea cucumber was commercially procured at a seafood market (Qingdao, China). The extraction and purification of Fuc-*Pg* were described in our previous study (15). The structure of Fuc-*Pg* used in the present study was consistent with our earlier research and was composed exclusively of fucose with a repetitive structure of [3Fuc(2S,4S)α1 → 3Fucα1 → 3Fuc(4S)α1 → 3Fucα1]n (13).

### 2.2 Animal experimental design

Thirty-two male ApoE^−/−^ mice and eight male C57BL/6 J mice (aged 6–7 weeks) obtained from Beijing Vital River Laboratory Animal Technology Co., Ltd. were maintained under standard conditions (room temperature, a 07:00–19:00 light–dark cycle, and ad libitum access to food and water). After one week of acclimatization, thirty-two ApoE^−/−^ mice were randomly distributed into four groups: M group (model group); S group (simvastatin group), which received 20 mg/kg simvastatin; the Fuc-*Pg*-L group (low-dose fucoidan group), which received 20 mg/kg fucoidan; and the Fuc-*Pg*-H group (high-dose fucoidan group), which received 80 mg/kg fucoidan (Figure 1A). C57BL/6 J mice were fed a normal diet and served as the control group (C group). ApoE^−/−^ mice were fed an HFD (42% kcal fat and 1.5% g/kg cholesterol). All interventions were administered by oral gavage once daily. The diet information was available in Supplementary Table 2. The food intake and body weight were recorded daily. After 12 weeks, all mice were fasted overnight and anesthetized; blood and tissues, including the liver, aorta, and small intestine, were then collected immediately after sacrifice. After complete isolation of the aorta from the mice, the surrounding adipose tissue was carefully dissected under a stereomicroscope. All samples were stored at −80 °C for further study. The procedures were permitted by the Animal Ethics Committee of Qingdao University (No. QDU-AEC-2023429).

**Figure 1:**
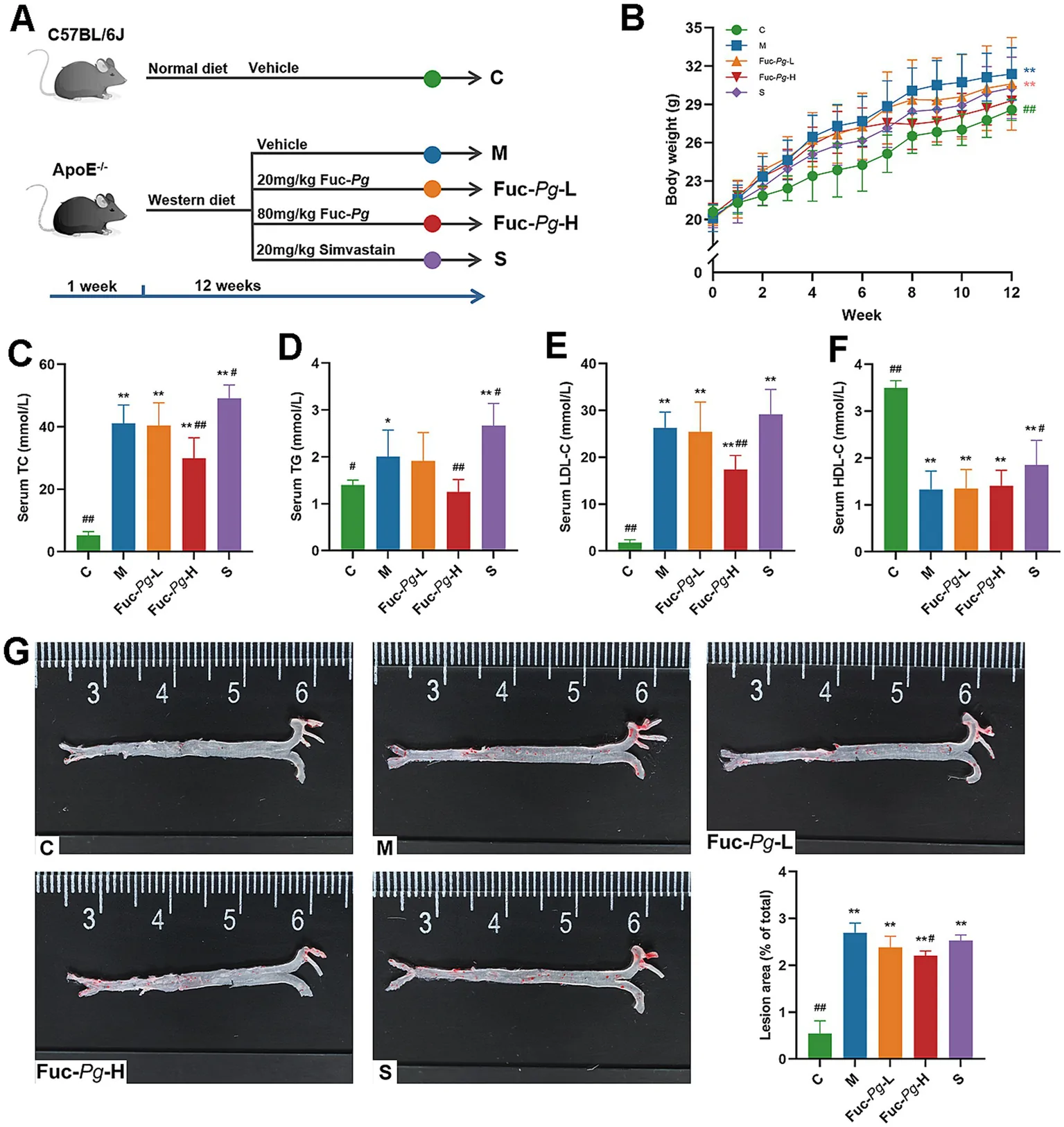
Fuc-*Pg* improved dyslipidemia and prevented AS in ApoE^−/−^ mice. **(A)** Diagram of the experimental design. **(B)** Body weight, *n* = 8. Serum levels of TC **(C)**, TG **(D)**, LDL-C **(E)**, and HDL-C **(F)**, *n* = 8. **(G)** Oil Red O staining. *p < 0.05 compared with the C group; **p < 0.01 compared with the C group; ^#^*p* < 0.05 compared with the M group; ^##^*p* < 0.01 compared with the M group. Fuc-*Pg*, fucoidan from *Pearsonothuria graeffei*; AS, atherosclerosis; TC, total cholesterol; TG, triglyceride; LDL-C, low-density lipoprotein cholesterol; HDL-C, high-density lipoprotein cholesterol; C, control group; M, model group.

### 2.3 Biochemistry assays

After homogenization and centrifugation of the frozen fecal samples and liver tissues, the supernatants were collected for measurement of biochemical indicators. Serum samples were retrieved from storage at −80 °C and allowed to equilibrate to room temperature prior to the measurements. The concentrations of TBA, TC, TG, LDL-C, and HDL-C were measured by enzymatic assays in strict accordance with the manufacturer’s instructions. Serum levels of TNF-α, IL-1β, and MCP-1 were quantified using ELISA, with all experimental steps performed according to the kit protocol.

### 2.4 Histology analysis

The complete aortic structure was excised from mice and preserved in 4% paraformaldehyde for 24 h. After meticulously excising the surplus perivascular adipose tissue, the aorta was longitudinally sectioned, stained with Oil Red O, and then imaged. The percentage of plaque area relative to the total aortic area was quantified by Image-Pro Plus 6.0.

### 2.5 Bile acid analysis

Serum samples (30 μL) were mixed with methanol (150 μL) to precipitate protein and extract bile acids. The mixtures were subjected to vortexing for 3 min and incubated at −20 °C for 1 h. Subsequently, the bile acid extracts were obtained by two cycles of high-speed centrifugation. Frozen liver samples (50 mg) were homogenized in 250 μL phosphate-buffered saline (PBS). After centrifugation, the supernatant was subjected to vortexing with five volumes of methanol and incubated at −20 °C for 1 h. Subsequently, the bile acid extracts were obtained by centrifugation. Bile acid analysis was performed using an Agilent 6,530 quadrupole time-of-flight (Q-TOF) instrument. Chromatographic separation was performed on a C18 column.

### 2.6 RT-qPCR

Total RNA from tissues was extracted using Trizol Reagent (Yeasen, Shanghai, China). The removal of genomic DNA and reverse transcription reactions were performed with cDNA Synthesis SuperMix (Yeasen, Shanghai, China). Reverse transcription-quantitative polymerase chain reaction (RT-qPCR) assays were performed using a QuantStudio 1 (Thermo Fisher Scientific Co., Ltd., Shanghai) with a SYBR Green Master Mix kit (Yeasen, Shanghai, China). Comprehensive experimental protocols for RT-qPCR have been described in an earlier publication (14). The target mRNA was normalized to glyceraldehyde-3-phosphate dehydrogenase (GAPDH) using the 2^−(ΔΔCT)^ method, with primer sequences detailed in Supplementary Table 3.

### 2.7 Western blot

The western blotting protocols were consistent with those used in a previous study (14). Liver and small intestinal tissue lysates were mixed with loading dye for sodium dodecyl sulfate-polyacrylamide gel electrophoresis (SDS-PAGE) and heat-denatured for analysis. The proteins were separated by SDS-PAGE and transferred onto polyvinylidene fluoride (PVDF) membranes. Following blocking with 5% skimmed milk, the membranes were incubated overnight with primary antibodies and treated with secondary antibodies for 1 h. Finally, protein blots were detected using chemiluminescence and quantified with ImageJ to determine their gray values.

### 2.8 Statistical analysis

Statistical analysis was performed in SPSS 27.0 using one-way analysis of variance (ANOVA) with Dunnett’s t-test. All data were presented as the mean ± standard deviation (SD). Statistical significance was set at *p* < 0.05.

## 3 Results

### 3.1 Fuc-*Pg* ameliorated dyslipidemia and reduced aortic plaque in ApoE^−/−^ mice

Initially, the mice had similar body weights (20.27 ± 0.80 g) across all groups (Figure 1B). After 12 weeks of feeding, the body weight of the M group (31.39 ± 2.06 g) was significantly higher than that of the C group (28.59 ± 0.83 g, *p* < 0.01). The two Fuc-*Pg* groups and the S group showed terminal body weights of 30.60 ± 3.62 g, 29.30 ± 1.10 g, and 30.29 ± 2.42 g, respectively. Relative to the C group, the M group presented hallmark features of dyslipidemia after long-term HFD feeding, demonstrating dramatically elevated TC, TG, and LDL-C concentrations and decreased HDL-C (*p* < 0.05, Figures 1C–F). Fuc-*Pg*-H markedly attenuated this pathological condition by reducing TC, TG, and LDL-C concentrations (*p* < 0.01), with a greater effect than Fuc-*Pg*-L. Simvastatin intervention did not alleviate dyslipidemia in ApoE^−/−^ mice. Only the HDL-C concentration in the S group was significantly higher than that in the M group (*p* < 0.05). The Oil Red O staining experiment visually revealed the protective effect of Fuc-*Pg* on atherosclerosis (Figure 1G). Compared with the C group, the M group showed heterogeneous red plaques from the aortic arch to the abdominal aorta. The aortic plaque areas were decreased in the two Fuc-*Pg* groups and the simvastatin group versus the M group. The reductions in arterial plaques in the S and Fuc-*Pg*-L groups were not statistically significant (*p* > 0.05). Fuc-*Pg*-H significantly attenuated this pathological manifestation, with a notable reduction in plaque in the aortic arch (*p* < 0.05). In summary, Fuc-*Pg* could delay the progression of atherosclerosis to a certain degree.

### 3.2 Fuc-*Pg* reduced serum and liver inflammation in ApoE^−/−^ mice

Inflammation is responsible for the progression of AS (16). Our previous study showed that Fuc-*Pg* could inhibit inflammation caused by HFD in C57BL/6 J mice (14). In this study, the M group presented higher concentrations of serum IL-1β, TNF-α, and MCP-1 than the C group (Figures 2A–C, *p* < 0.01). Fuc-*Pg* intervention substantially reduced the values of serum inflammatory factors compared with the M group (*p* < 0.01), yet these values remained above those in the C group (*p* < 0.01). The M group also showed distinctly higher hepatic mRNA levels of IL-1β and TNF-α versus the C group (*p* < 0.05, *p* < 0.01, Figures 2D–F), while MCP-1 expression exhibited a non-significant increase (*p* > 0.05). Fuc-*Pg*-H decreased these pro-inflammatory factors relative to the M group (*p* < 0.05 or *p* < 0.01). Simvastatin administration failed to demonstrate any significant impact on serum and liver inflammatory factors versus the M group. These findings demonstrate that Fuc-*Pg* could alleviate the systemic inflammatory state in ApoE^−/−^ mice fed an HFD.

**Figure 2:**
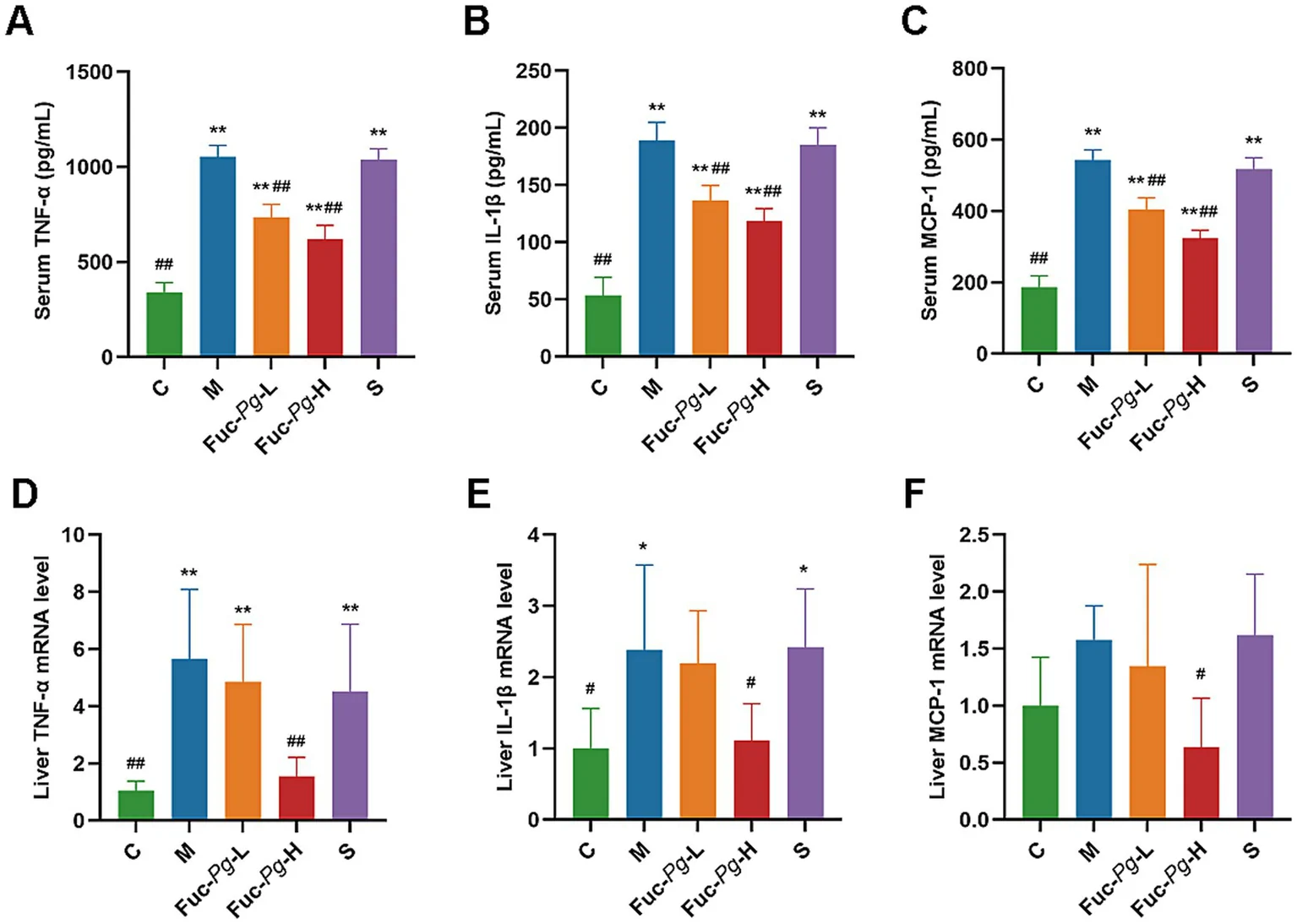
Fuc-*Pg* improved serum and liver inflammation in ApoE^−/−^ mice. Serum levels of TNF-α **(A)**, IL-1β **(B)**, and MCP-1 **(C)**, *n* = 8. The mRNA levels of hepatic TNF-α **(D)**, IL-1β **(E)**, and MCP-1 **(F)**, *n* ≥ 4. *: *p* < 0.05 compared with the C group; **p < 0.01 compared with the C group; ^#^*p* < 0.05 compared with the M group; ^##^*p* < 0.01 compared with the M group. Fuc-*Pg*, fucoidan from *Pearsonothuria graeffei*; TNF-α, tumor necrosis factor-α; IL-1β, interleukin-1β; MCP-1, monocyte chemoattractant protein-1; mRNA, messenger RNA; C, control group; M, model group.

### 3.3 Fuc-*Pg* inhibited intestinal lipid absorption and promoted lipid efflux in ApoE^−/−^ mice

Lipids absorbed by enterocytes are assembled into chylomicrons and subsequently transported throughout the body via the lymphatic system. An elevated rate of chylomicron formation can result in dyslipidemia, thereby heightening the risk of atherosclerosis (17). We assessed the protein expression levels of CD36 and microsomal triglyceride transfer protein (MTP) in the duodenum, which are crucial for the assembly and secretion of chylomicrons. In Figures 3A–C, duodenal CD36 and MTP protein levels in the M group were considerably elevated compared with those in the C group (*p* < 0.01). Fuc-*Pg*-H substantially suppressed the protein levels of CD36 and MTP relative to the M group (*p* < 0.01 and *p* < 0.05). The S group demonstrated a decline in MTP protein expression versus the M group (*p* < 0.05), while CD36 protein expression remained unaltered (*p* > 0.05). ABCG5 in the distal small intestine is closely related to cholesterol absorption as a cholesterol efflux transporter (18). There was almost no difference in ileal ABCG5 protein level between the C and M groups. Relative to the M group, the ileal ABCG5 protein level in the Fuc-*Pg* intervention groups was higher, but the difference was not significant (Figure 3D, *p* > 0.05). We subsequently measured the concentrations of fecal TC and TG (Figures 3E,F). Relative to the C group, fecal TC and TG levels in ApoE^−/−^ mice fed an HFD were higher, but the differences were not significant (*p* > 0.05). Fuc-*Pg* at both dosages significantly enhanced the excretion of fecal TC and TG relative to the C group (*p* < 0.01). Fuc-*Pg*-H markedly elevated the TG content versus the M group (*p* < 0.01). Simvastatin intervention partly increased the excretion of fecal lipid versus the C group (*p* < 0.05). These findings confirmed that Fuc-*Pg* might decrease lipid absorption by inhibiting the assembly and secretion of chylomicrons.

**Figure 3:**
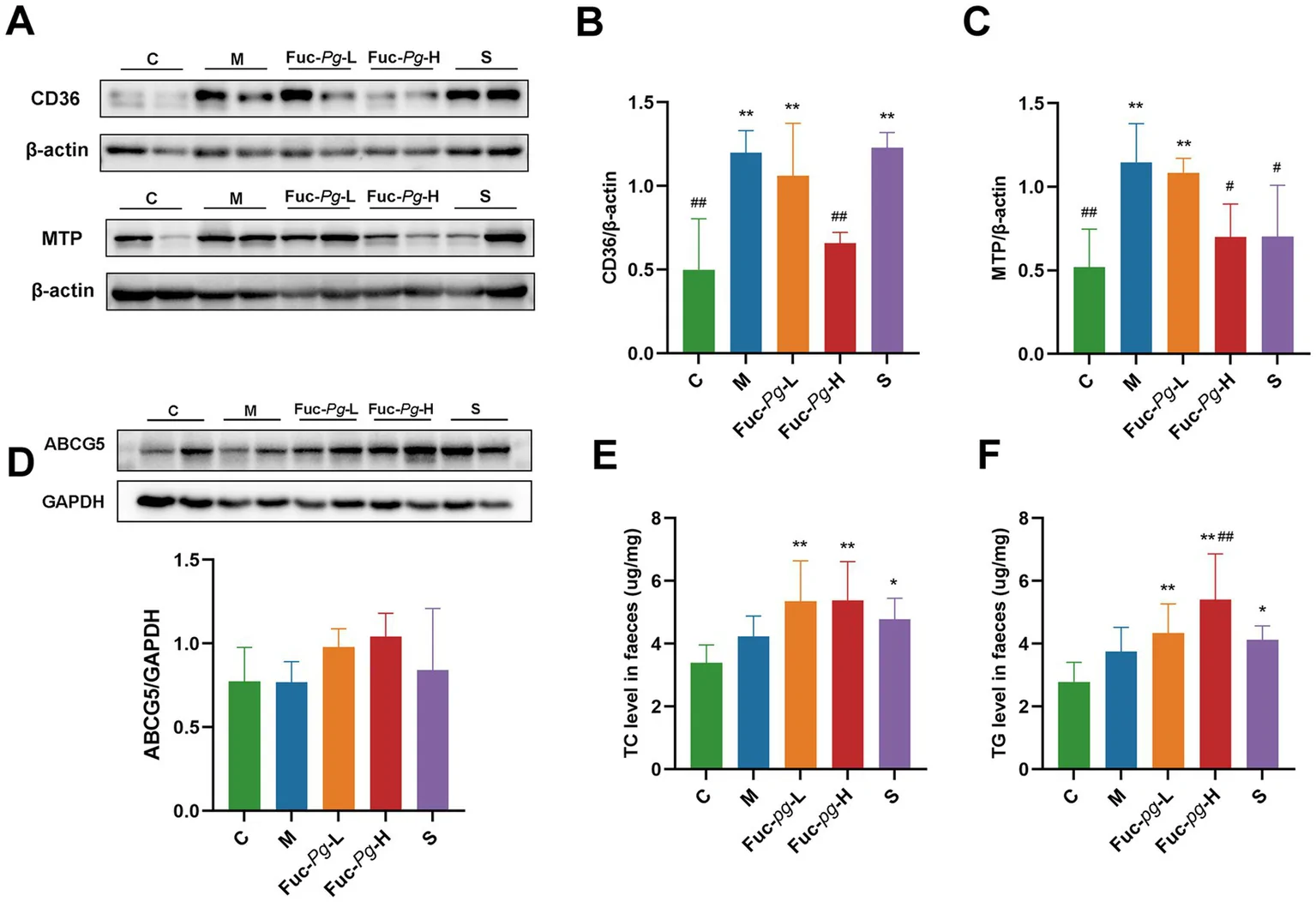
Fuc-*Pg* regulated lipid metabolism in the small intestine. **(A)** Western blotting results of CD36 and MTP protein expression in the duodenum, *n* ≥ 4. **(B,C)** Relative intensities of CD36 and MTP calculated after normalization against β-actin. **(D)** Western blot assay of the protein level of ABCG5 in the ileum, *n* ≥ 4. **(E,F)** TC and TG levels in feces, *n* ≥ 4. *p < 0.05 compared with the C group; **p < 0.01 compared with the C group; ^#^*p* < 0.05 compared with the M group; ^##^*p* < 0.01 compared with the M group. Fuc-*Pg*, fucoidan from *Pearsonothuria graeffei*; MTP, microsomal triglyceride transfer protein; ABCG5, ATP-binding cassette protein G5; TC, total cholesterol; TG, triglyceride; C, control group; M, model group.

### 3.4 Fuc-*Pg* alleviated disorders of hepatic cholesterol metabolism in ApoE^−/−^ mice

#### 3.4.1 Fuc-*Pg* enhanced reverse cholesterol transport (RCT) in ApoE^−/−^ mice

AS development is implicated in the disruption of hepatic lipid metabolism, particularly abnormal cholesterol metabolism (19). In Figure 4A, the M group had a notably heavier liver weight than the C group (*p* < 0.01). There was no significant difference in liver weight between the C and Fuc-*Pg*-H groups (*p* > 0.05). The Fuc-*Pg*-L and S groups exhibited substantially higher liver TC content relative to the C group (*p* < 0.01, Figure 4B). Despite the liver TC content of the Fuc-*Pg*-H group being slightly higher than that in the C group, this difference was non-significant (*p* > 0.05). Therefore, Fuc-*Pg* might alleviate the disorder of liver cholesterol metabolism.

**Figure 4:**
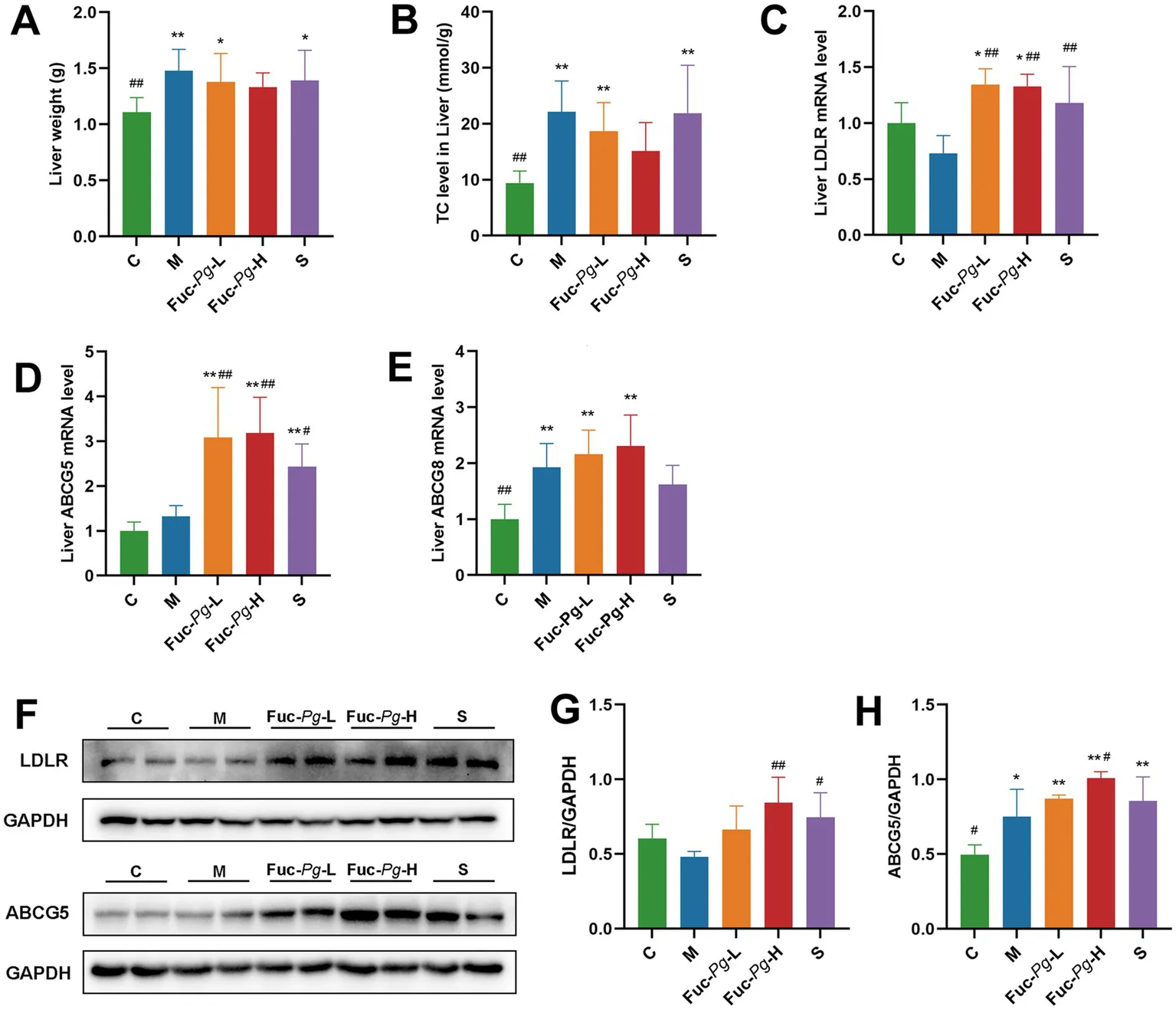
Fuc-*Pg* regulated hepatic cholesterol metabolism in ApoE^−/−^ mice. **(A)** Liver weight, *n* ≥ 8. **(B)** TC level in liver, *n* ≥ 8. **(C–E)** The mRNA levels of genes related to hepatic LDLR, ABCG5, and ABCG8, *n* ≥ 4. **(F)** Western blotting results of LDLR and ABCG5 protein expression in the liver, *n* ≥ 4. **(G,H)** Relative intensities of LDLR and ABCG5 were calculated after normalization against GAPDH. *p < 0.05 compared with the C group; **p < 0.01 compared with the C group; ^#^*p* < 0.05 compared with the M group; ^##^*p* < 0.01 compared with the M group. Fuc-*Pg*, fucoidan from *Pearsonothuria graeffei*; TC, total cholesterol; LDLR, low-density lipoprotein receptor; ABCG5/8, ATP-binding cassette proteins G5/8; GAPDH, glyceraldehyde-3-phosphate dehydrogenase; C, control group; M, model group.

Reverse cholesterol transport (RCT) refers to the process by which surplus cholesterol in peripheral tissues is delivered to the liver for subsequent elimination via biliary secretion or conversion into bile acids. Enhanced RCT is beneficial for eliminating excess cholesterol, thereby ameliorating AS (20). We examined the relevant genes and proteins in the liver to explore whether Fuc-*Pg* affects RCT. The M group presented a downward trend in LDLR mRNA levels versus the C group, albeit without statistical significance (Figure 4C, *p* > 0.05). Fuc-*Pg* considerably upregulated LDLR mRNA expression compared with the C group (*p* < 0.05) and the M group (*p* < 0.01). LDLR expression in the S group was significantly elevated at the mRNA level versus the M group (*p* < 0.01). The mRNA levels of cholesterol efflux mediators ABCG5 and ABCG8 were elevated in the M group versus the C group (Figures 4D,E), with the upregulation of ABCG8 reaching statistical significance (*p* < 0.01). Following intervention with Fuc-*Pg*, the mRNA levels of the two genes markedly increased relative to the C group (*p* < 0.01). Both Fuc-*Pg* administration groups showed higher ABCG5 and ABCG8 mRNA levels relative to the M group, and the elevation in ABCG5 reached statistical significance (*p* < 0.01). Likewise, notably higher levels of the two genes in the S group were detected versus the C group (*p* < 0.01). Relative to the M group, the S group had a markedly higher ABCG5 mRNA level (*p* < 0.05). We further confirmed the protein expression of LDLR and ABCG5 (Figures 4F–H). The M group had lower LDLR protein expression than the C group (*p* > 0.05). High-dose Fuc-*Pg* and simvastatin administration markedly elevated the LDLR protein level versus the M group (*p* < 0.01 and *p* < 0.05). ABCG5 protein expression was markedly increased in the four groups versus the C group (*p* < 0.05 or *p* < 0.01). The difference in ABCG5 protein expression among the Fuc-*Pg*-L, S, and M groups was not significant (*p* > 0.05). ABCG5 protein levels in the Fuc-*Pg*-H group were notably increased relative to the M group (*p* < 0.01). The findings suggested that Fuc-*Pg* administration affected RCT, especially through its effects on LDLR and ABCG5 expression.

#### 3.4.2 Fuc-*Pg* facilitated catabolism of cholesterol into bile acids in ApoE^−/−^ mice

It is essential for cholesterol to be converted into bile acids (BAs) in the liver to maintain systemic cholesterol homeostasis, which effectively contributes to the prevention of AS (21). In Figure 5A, Fuc-*Pg*-H considerably elevated the hepatic TBA level relative to the C and M groups (*p* < 0.05 and *p* < 0.01). Hepatic genes associated with bile acid synthesis were further verified. Peroxisome proliferator-activated receptor-gamma coactivator-1alpha (PGC1α) and hepatocyte nuclear factor 4 (HNF4α) synergistically regulate the expression of hepatic bile acid synthesis enzymes CYP7A1 and CYP27A1 (22). We observed that Fuc-*Pg*-H had higher mRNA levels of PGC1α (*p* < 0.01) and HNF4α (*p* < 0.05) versus the M group (Figures 5B,C). The Fuc-*Pg*-H group had higher mRNA levels of PGC1α and HNF4α than the C group, but the differences were not significant (*p* > 0.05). The HNF4α mRNA level was significantly different between the S and M groups (*p* < 0.05). Fuc-*Pg* administration markedly elevated CYP7A1 and CYP27A1 mRNA levels compared with the C and M groups (*p* < 0.01, Figures 5D,E). Fuc-*Pg* administration at both dosages markedly elevated CYP7A1 and CYP27A1 expression at the protein level versus the C group (*p* < 0.05 and *p* < 0.01, Figures 5F–H). The CYP7A1 protein level in the Fuc-*Pg*-H group was significantly elevated versus the M group (*p* < 0.05). Simvastatin intervention enhanced CYP7A1 expression at the mRNA level (*p* < 0.01), but it failed to improve CYP7A1 protein levels versus the M group (*p* > 0.05). Taken together, Fuc-*Pg* might promote bile acid synthesis by modulating the PGC1α/HNF4α signaling pathway to activate CYP7A1 and CYP27A1 expression.

**Figure 5:**
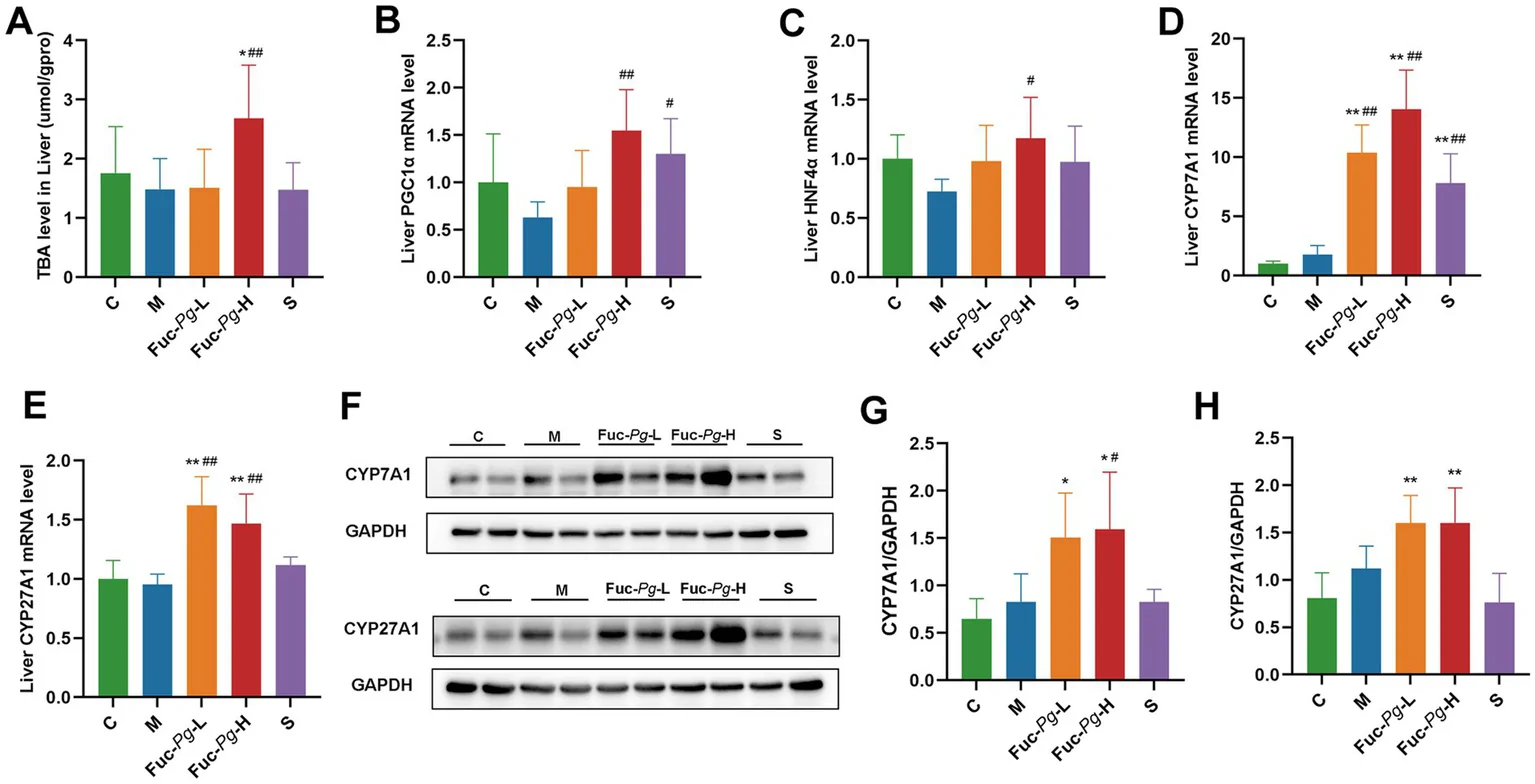
Fuc-*Pg* regulated hepatic bile acid metabolism in ApoE^−/−^ mice. **(A)** TBA levels in the liver, *n* = 8. **(B–E)** The mRNA levels of genes related to hepatic PGC1α, HNF4α, CYP7A1, and CYP27A1, *n* ≥ 4. **(F)** Western blotting results of CYP7A1 and CYP27A1 protein expression in the liver, *n* ≥ 4. **(G,H)** Relative intensities of CYP7A1 and CYP27A1 were calculated after normalization against GAPDH. *p < 0.05 compared with the C group; **p < 0.01 compared with the C group; ^#^*p* < 0.05 compared with the M group; ^##^*p* < 0.01 compared with the M group. Fuc-*Pg*, fucoidan from *Pearsonothuria graeffei*; TBA, total bile acid; PGC1α, peroxisome proliferator-activated receptor-gamma coactivator-1α; HNF4α, hepatocyte nuclear factor 4α; GAPDH, glyceraldehyde-3-phosphate dehydrogenase; C, control group; M, model group.

We then performed a comprehensive analysis using ultra-performance liquid chromatography-quadrupole time-of-flight mass spectrometry (UPLC-QTOF/MS) to explore the impact of Fuc-*Pg* on hepatic bile acid profiles. High-dose Fuc-*Pg* treatment showed upward trends in the levels of BAs, including cholic acid (CA), chenodeoxycholic acid (CDCA), alpha-muricholic acid (αMCA), beta-muricholic acid (βMCA), taurocholic acid (TCA), tauroursodeoxycholic acid (TUDCA), tauro-alpha-muricholic acid (TαMCA), tauro-beta-muricholic acid (TβMCA), and tauro-omega-muricholic acid (TωMCA) relative to the M group (Supplementary Figure 2). In particular, Fuc-*Pg*-H notably increased the TβMCA level relative to the M group (*p* < 0.01) and the C group (*p* < 0.05). An increased serum BA level in cases of liver damage serves as an important early warning signal. Considering the improved liver bile acid synthesis, we further analyzed the impact of Fuc-*Pg* on serum TBA and BA profiles. The differences in serum TBA levels and profiles among the five groups were not significant (Supplementary Figure 3). The M group exhibited a slight elevation in TBA levels compared to the C group. This elevated level was reduced following Fuc-*Pg* intervention, whereas simvastatin treatment did not affect TBA levels. Relative to the M group, Fuc-*Pg* treatment showed a downward trend in the levels of most BAs, except for TβMCA, which demonstrated a modest elevation. The trends toward increased hepatic bile acid levels and decreased serum bile acid levels suggested that Fuc-*Pg* regulated cholesterol metabolism by promoting bile acid synthesis with protective effects on the liver.

## 4 Discussion

Despite possessing limited commercial value due to their unpalatable properties, *Pearsonothuria graeffei* contains abundant functional ingredients, particularly polysaccharides and saponins (23, 24). The precise structural elucidation of Fuc-*Pg* facilitates its potential application as a bioactive constituent in high-purity and high-quantity preparations. In the present study, Fuc-*Pg* administration significantly improved serum dyslipidemia and reduced aortic plaques in ApoE^−/−^ mice fed an HFD. Fuc-*Pg* has been demonstrated to alleviate systemic inflammation through the inhibition of the Toll-like receptor 4/nuclear factor kappa B (TLR4/NF-κB) pathway in C57BL/6 J mice (14). Consistent with this, the present work confirms its ability to repress inflammation in ApoE^−/−^ mice by reducing liver and serum inflammatory factors.

Changes in dietary patterns in recent decades have emerged as significant health detriments. The intestinal tract is the primary site where lipids, including TG and cholesterol, are absorbed. These lipids are assembled into chylomicrons in the enterocytes and subsequently released into the blood circulation (25). Under normal dietary conditions, chylomicrons undergo rapid catabolism into remnant particles and then are utilized by organs or tissues without compromising systemic health. Atherogenic diets such as HFD could cause high serum levels of chylomicron remnants (26). Hence, reducing intestinal chylomicron production is a potential target for preventing chronic metabolic diseases (27). Our previous study found that Fuc-*Pg* reduced intestinal CD36 expression in C57BL/6 J mice fed an HFD (14). CD36, functioning as a fatty acid transporter, mediates lipid uptake and chylomicron packaging. In the present study, Fuc-*Pg* also reduced intestinal CD36 in ApoE^−/−^ mice fed an HFD. Meanwhile, Fuc-*Pg* was observed to downregulate MTP expression in intestinal epithelial cells. MTP is an important enzymatic protein involved in chylomicron production and facilitates the assembly of dietary lipids into pre-chylomicrons (28). Additionally, Fuc-*Pg* increased the ileal ABCG5 protein level. The ABCG5 transporter facilitates the reverse transport of absorbed cholesterol from enterocytes back to the gut lumen, thereby reducing cholesterol absorption in the distal small intestine (18). Elevated fecal cholesterol and TG concentrations verified that Fuc-*Pg* may impede lipid absorption and promote lipid efflux.

The liver, the largest digestive gland in the human body, maintains cholesterol metabolic balance through hepatocytes that transform, transport, and excrete cholesterol via multiple metabolic pathways. Some researchers have demonstrated that upregulating the expression of LDLR and ABCG5 transporters enhances RCT and attenuates AS (29, 30). LDLR is situated on the surface of hepatocytes, where it mediates the endocytosis of LDL-C in the circulation, thereby removing over 70% of circulating LDL particles (30). Cholesterol entering hepatocytes undergoes metabolism via multiple pathways: a portion is utilized to meet the cells’ metabolic requirements, a portion is excreted into bile via the ABCG5/ABCG8 transporters, and another portion is converted to bile acids for elimination (31). Fuc-*Pg* intervention upregulated LDLR and ABCG5/ABCG8 expression, thereby reducing hepatic lipid deposition. Abnormal cholesterol metabolism in the liver not only directly impairs hepatic function, resulting in conditions such as fatty liver and cirrhosis, but also induces dyslipidemia, thereby elevating the risk of AS and cardiovascular diseases (32). Our results support the notion that Fuc-*Pg* could alleviate AS by regulating the RCT pathway.

The high hepatic TBA concentration indicated that Fuc-*Pg* might also favor the conversion of cholesterol into bile acids. There are two pathways involved in bile acid synthesis: the classic pathway initiated by CYP7A1 and the alternative pathway initiated by CYP27A1 (33). Our data showed that Fuc-*Pg* enhanced bile acid synthesis pathways. HNF4α, a nuclear receptor and a central regulator of hepatic gene expression, acts as a primary positive regulator of CYP7A1 transcription. By directly interacting with HNF4α, PGC1α significantly enhances its transcriptional activity to upregulate the expression of CYP7A1. In addition, HNF4α can bind to the promoter region of CYP27A1 to enhance its transcriptional activity (34, 35). Through a series of enzymatic reactions, cholesterol is ultimately converted into CA and CDCA. CDCA undergoes metabolic conversion into MCAs through 6β-hydroxylation, including αMCA and βMCA. Most BAs are conjugated with taurine and subsequently excreted into bile (36). TUDCA has been demonstrated to protect hepatocytes from injury through mechanisms including stabilizing cell membranes, alleviating endoplasmic reticulum stress, and inhibiting the mitochondria-dependent apoptotic cascade (37). TβMCA is hydrophilic, and its increased levels help reduce the toxicity of hydrophobic bile acids to hepatocytes (38). Consistent with the effects of Fuc-*Pg*, seaweed-derived fucoidan has been shown to promote the conversion of cholesterol to BAs by upregulating CYP7A1 (1).

However, there are several limitations in the present study. First, despite the use of a classic animal model in this study, there remain notable differences in pathological features between the model and human diseases, potentially limiting the clinical applicability of the results. Second, the positive drug control used in the present study partly failed to improve blood lipid profiles in ApoE-deficient mice, which is consistent with previous research (39). Given that simvastatin lowers lipids primarily by inhibiting 3-hydroxy-3-methylglutaryl coenzyme A (HMG-CoA) reductase and thereby suppressing endogenous cholesterol biosynthesis (40), its efficacy may be limited in the presence of a high dietary fat load. In addition, the lipid-clearing capacity of simvastatin potentially depends on the functional integrity of ApoE protein (41). Furthermore, the plaque-stabilizing capacity of statins has been proven to operate independently of their lipid-modulating effects, but these benefits may require prolonged intervention to manifest (42). Therefore, other drugs such as ezetimibe should be selected as positive controls for studies using ApoE-deficient mice.

## 5 Conclusion

Our study confirmed that Fuc-*Pg* alleviated the lipid disorder in ApoE^−/−^ mice fed an HFD. Further studies demonstrated that Fuc-*Pg* reduced serum and hepatic cholesterol levels by regulating intestinal lipid metabolism, enhancing hepatic RCT, and facilitating cholesterol bioconversion into bile acids for elimination. This research provides a valuable foundation for exploring functional food ingredients aimed at alleviating AS.

## Data Availability

The original contributions presented in the study are included in the article/Supplementary material, further inquiries can be directed to the corresponding author.

## Glossary

Fuc-*Pg*: Fucoidan from *Pearsonothuria graeffei*
RCT: Reverse cholesterol transport
HFD: High-fat diet
AS: Atherosclerosis
TLRs: Triglyceride-rich lipoproteins
PCSK9: Proprotein convertase subtilisin/kexin type 9
TG: Triglyceride
TC: Total cholesterol
LDL-C: Low-density lipoprotein cholesterol
HDL-C: High-density lipoprotein cholesterol
TBA: Total bile acid
TNF-α: tumor necrosis factor α
IL-1β: Interleukin-1β
MCP-1: Monocyte chemoattractant Protein-1
1H NMR: One-dimensional hydrogen nuclear magnetic resonance
MTP: Microsomal triglyceride transfer protein
ABCG5/8: ATP-binding cassette protein G5/8
LDLR: Hepatic low-density lipoprotein receptor
PGC1α: Peroxisome proliferator-activated receptor-gamma coactivator-1alpha
HNF4α: Hepatocyte nuclear factor 4
CA: Cholic acid
CDCA: Chenodeoxycholic acid
αMCA: Alpha-muricholic acid
βMCA: Beta-muricholic acid
TCA: Turocholic acid
TUDCA: Tauroursodeoxycholic acid
TαMCA: Tauro-alpha-muricholic acid
TβMCA: Tauro-beta-muricholic acid
TωMCA: Tauro-omega-muricholic acid
