# Supplementary material for: Fucoidan from Pearsonothuria graeffei alleviates dyslipidemia in ApoE−/− mice by regulating reverse cholesterol transport and promoting bile acid synthesis
Source: Front Nutr. 2026 Oct 19;13:1974149. doi: 10.3389/fnut.2026.1974149 (PMC13724345; doi:10.3389/fnut.2026.1974149)
Supplement: Supplementary file 1 [file Supplementary_file_1.docx]

Fucoidan from *Pearsonothuria graeffei* Alleviates Dyslipidemia in ApoE^-/-^ Mice by Regulating Reverse Cholesterol Transport and Promoting Bile Acid Synthesis

Ting Zhao^1 †^, Yang Yu^2 †^, Yin Wang^3^, Liyang Zhang^3^, Junqi Xiao^3^, Shiguo Chen^4^ * and Shan Li^3^,*

Supplementary table 1 Bile acid standards information

| Bile acids | Manufacturers |
| --- | --- |
| Cholic acid (CA) | TargetMol (Wellesley Hills, United States) |
| Deoxycholic acid (DCA) | TargetMol (Wellesley Hills, United States) |
| Ursodeoxycholic acid (UDCA) | TargetMol (Wellesley Hills, United States) |
| Chenodeoxycholic acid (CDCA) | TargetMol (Wellesley Hills, United States) |
| Taurocholic acid (TCA) | TargetMol (Wellesley Hills, United States) |
| Taurochenodeoxycholic acid (TCDCA) | TargetMol (Wellesley Hills, United States) |
| Tauroursodeoxycholic acid (TUDCA) | TargetMol (Wellesley Hills, United States) |
| Alpha-muricholic acid (α-MCA) | Yuanye Bio-Technology (Shanghai, China) |
| Beta-muricholic acid (β-MCA) | Yuanye Bio-Technology (Shanghai, China) |
| Omega-muricholic acid (ω-MCA) | Yuanye Bio-Technology (Shanghai, China) |
| Tauro-alpha-muricholic acid (T-α-MCA) | Yuanye Bio-Technology (Shanghai, China) |
| Tauro-beta-muricholic acid (T-β-MCA) | Yuanye Bio-Technology (Shanghai, China) |
| Tauro-omega-muricholic acid (T-ω-MCA) | Yuanye Bio-Technology (Shanghai, China) |

Supplementary table 2 Ingredient Composition of the Experimental Diets

| Ingridient | Normal diet (g/kg) | HFD (g/kg) |
| --- | --- | --- |
| Protein(casein, L-cystine) | 155.00 | 195.00 |
| Carbohydrate(starch, sucrose) | 701.00 | 476.00 |
| Fat(corn oil, milkfat) | 50.00 | 210.00 |
| Cellulose | 48.00 | 60.00 |
| Mineral mix and vitamin mix | 43.00 | 54.00 |
| Choline supplement | 2.00 | 3.00 |
| Cholesterol | 0.00 | 1.50 |
| TBHQ | 0.01 | 0.04 |

Supplementary table 3 The primers sequences used for Rt-qPCR

| Gene | Forward primer sequence (5'-3') | Reverse primer sequence (5'-3') |
| --- | --- | --- |
| Gapdh | CCCATGTTTGTGATGGGTGT | GTGATGGCATGGACTGTGGT |
| Abcg5 | TTCGGTCACGGGCACAGAGG | TGGTCTTCCCTGAGCCTGAGC |
| Abcg8 | GCTCGTGTGGTTGGTGGTCTTC | GCCGTAGCTGATGCCGATGAC |
| Ldlr | GCTTGGACAAGGTGTGCGACTC | CGTGGCGGTTGGTGAAGAGC |
| Cyp7a1 | TGTCTGGGTCACGGAAGGGATG | GGATCACGAGGTGCGTCTTAGC |
| Cyp27a1 | AAGGACCACCGAGACCACAAGG | GGAGGGTTTCAGGCAGCCAATC |
| MCP-1 | CCACTCACCTGCTGCTACTCATTC | GTTCACTGTCACACTGGTCACTCC |
| IL-1β | TGGAAAAGCGGTTTGTCTTC | TACCAGTTGGGGAACTCTGC |
| TNFα | CACCACGCTCTTCTGTCTACTGAAC | TGACGGCAGAGAGGAGGTTGAC |
| Pgc1α | ACGATGACCCTCCTCACACCAA | ATGTTGCGACTGCGGTTGTGTA |
| Hnf4α | GCCTTCTGCGAACTCCTTCTGG | CAGTGCCGAGGGACGATGTAGT |

Supplementary Figure 1 (A) Average daily food intake. (B) Average daily energy intake. ^*^: *P* < 0.05 compared with the C group; ^**^: *P* < 0.01 compared with the C group; ^#^: *P* < 0.05 compared with the M group; ^##^: *P* < 0.05 compared with the M group.

Supplementary Figure 2 Fuc-*Pg* changed hepatic bile acids levels in ApoE^-/-^ mice. Hepatic levels of CA (A), CDCA (B), UDCA (C), αMCA (D), βMCA (E), ωMCA (F), TCA (G), TCDCA (H), TUDCA (I), TαMCA (J), TβMCA (K) and TωMCA (L). *: *P* < 0.05 compared with the C group; **: *P* < 0.01 compared with the C group; #: P < 0.05 compared with the M group; ##: *P* < 0.01 compared with the M group.

Supplementary Figure 3 Fuc-*Pg* changed serum bile acids levels in ApoE*^-/-^* mice. (A) Serum TBA level. Serum levels of CA (B), CDCA (C), DCA (D), βMCA (E), ωMCA (F), TCA (G), TCDCA (H), TUDCA (I), TβMCA (J) and TωMCA (K).
